# Reply to commentary on costs of diabetes and its complications in Poland

**DOI:** 10.1007/s10198-014-0661-x

**Published:** 2014-12-09

**Authors:** Joanna Leśniowska

**Affiliations:** Department of Economics, Kozminski University, Warsaw, Poland

Thank you very much for the comments on my article. I am glad that the paper has raised and contributed to the international debate on the costs of diabetes.

Indeed, the calculated costs do not control for the stage of the disease. However, I could not have proceeded that way as the Polish National Health Fund (NHF) does not split the cost data by the severity of a given disorder. This is mainly due to the fact that individual primary health care providers are not required to report the values of services provided. Therefore, only the number of services is available. According to the published NHF data, as many as 5,469,444 diabetes patients were advised within the primary care system accounting for 14.45 % of the total patients counseling. Due to the lack of detailed and accurate data on the cost of primary care, they can only be estimated on the NHF reports’ basis.

**Fig. 1:**
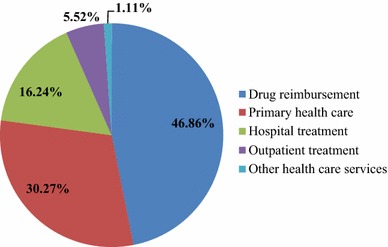
The structure of the direct costs of diabetes in 2012

It should be noted, however, that the structure of the direct costs reflects—to a certain extent—the severity of diabetes. Analyzing the structure of these costs in 2012, we can observe that the largest percentage of them is generated by the drug reimbursement and primary care costs (see Fig. 1).

It is worth notifying that at the moment Poland lacks access to innovative medicines. The Ministry of Health has not agreed to reimburse the incretin treatment. Finally, the financing scheme for health care providers needs to be changed. The costs-reduction principle may lead health providers to amputate the limb instead of treating the diabetic foot.

